# Accuracy of Digital Imaging Software to Predict Soft Tissue Changes during Orthodontic Treatment

**DOI:** 10.3390/jimaging10060134

**Published:** 2024-05-31

**Authors:** Theerasak Nakornnoi, Pannapat Chanmanee

**Affiliations:** 1 Department of Orthodontics, Faculty of Dentistry, Mahidol University, Ratchathewi, Bangkok 10400, Thailand; 2 Orthodontic Section, Department of Preventive Dentistry, Faculty of Dentistry, Prince of Songkla University, Hat Yai, Songkhla 90110, Thailand

**Keywords:** conventional orthodontics, Digital Imaging software, orthognathic surgery, soft tissue prediction

## Abstract

This study aimed to evaluate the accuracy of the Digital Imaging software in the prediction of soft tissue changes following three types of orthodontic interventions: non-extraction, extraction, and orthognathic surgery treatments. Ninety-six patients were randomly selected from the records of three orthodontic interventions (32 subjects per group): (1) non-extraction, (2) extraction, and (3) orthodontic treatment combined with orthognathic surgery. The cephalometric analysis of soft tissue changes in both the actual post-treatment and the predicted treatment was performed using Dolphin Imaging software version 11.9. A paired *t*-test was utilized to assess the statistically significant differences between the predicted and actual treatment outcomes of the parameters (*p* < 0.05). In the non-extraction group, prediction errors were exhibited only in the lower lip parameters. In the extraction group, prediction errors were observed in both the upper and lower lip parameters. In the orthognathic surgery group, prediction errors were identified in chin thickness, facial contour angle, and upper and lower lip parameters (*p* < 0.05). Digital Imaging software exhibited inaccurate soft tissue prediction of 0.3–1.0 mm in some parameters of all treatment groups, which should be considered regarding the application of Dolphin Imaging software in orthodontic treatment planning.

## 1. Introduction

Facial soft tissue plays a pivotal role in facial aesthetics [1], which constitutes a significant objective in contemporary orthodontic treatments and is often a primary motivation for patients seeking orthodontic care. Variations in soft tissue changes were observed across different age groups, genders, treatment approaches, and pre-treatment soft tissue characteristics [2]. Moreover, predicting a post-treatment soft tissue profile change is a complex task that necessitates consideration of various variables to account for the variability [2]. Therefore, orthodontists encounter significant challenges in forecasting soft tissue outcomes; however, accurate soft tissue prediction is crucial in clinical practice.

Previous manual treatment prediction using cephalometric tracing methods is susceptible to errors in the identification of anatomical landmarks and demands more time for measurement [3]. Consequently, the integration of contemporary digital software aims to overcome the limitations of conventional cephalometric tracing. Computer-assisted cephalometric prediction software, such as the Dolphin Imaging system, offers significant advantages over traditional methods by enabling rapid measurements shortly after marking anatomical landmarks on the radiograph. This not only minimizes the time and effort invested by the orthodontist in treatment planning but also eliminates measurement errors [4,5]. Therefore, digital software has witnessed increased utilization for visual simulation and the prediction of orthodontic treatment outcomes.

Digital prediction software forecasts treatment outcomes by superimposing the patient’s lateral cephalogram and lateral profile photographs. The program represents the expected treatment results in accordance with orthodontic interventions. This information facilitates valuable comparisons among diverse treatment approaches [6,7] that can enable orthodontists to effectively communicate treatment overviews and expected outcomes to patients [8]. However, inaccuracies in simulating soft tissue changes persist with these software applications. Consequently, it is essential to delineate the magnitude, direction, and location of such errors to comprehensively evaluate their potential clinical implications [9].

The accuracy of predictions of soft tissue changes plays a crucial role in orthodontic treatment planning. Although numerous studies have investigated the accuracy and reliability of prediction software, the outcomes have often been inconsistent [10,11,12]. Most of these inaccuracies and disputes are specifically associated with soft tissue predictions. The final outcomes typically deviate from the simulated image due to individual variations in soft tissue adaptation to accommodate skeletal and dental alterations [7]. Furthermore, variations in treatment modalities have been shown to significantly impact these soft tissue responses [2,13]. The potential inaccuracy in soft tissue prediction can lead to unrealistic patient expectations and subsequent dissatisfaction with post-treatment outcomes. Consequently, the aim of this study was to assess the accuracy of Digital Imaging software in predicting soft tissue changes following orthodontic treatment, encompassing cases of non-extraction, extraction, and orthodontic treatment combined with orthognathic surgery. This evaluation compared the discrepancies between the predicted and actual values of these changes.

## 2. Materials and Methods

This study was designed as a retrospective, cross-sectional observational study. All orthodontic patients from the dental hospital at the Faculty of Dentistry, Prince of Songkla University, who completed their treatment and fulfilled the inclusion criteria were randomly enrolled in this study. This study was approved by the institutional human research ethics committee (No. EC6502-005) and conducted in accordance with the Declaration of Helsinki.

### 2.1. Subject Selection

Determination of the sample size was conducted by referencing a previous study [10] using the G*Power program version 3.1 (Heinrich-Heine-Universität Düsseldorf, Düsseldorf, Germany), with a significance level set at 0.05 and a study power of 80%. A minimum of 32 subjects per group was required. All subjects were randomly selected from three categories based on the treatment modalities: (1) non-extraction, (2) extraction, and (3) orthodontic treatment combined with orthognathic surgery. As a result, a total of 96 subjects were included in this study. The inclusion and exclusion criteria are shown in Table 1.

### 2.2. Cephalometric Analysis

**Figure 1:**
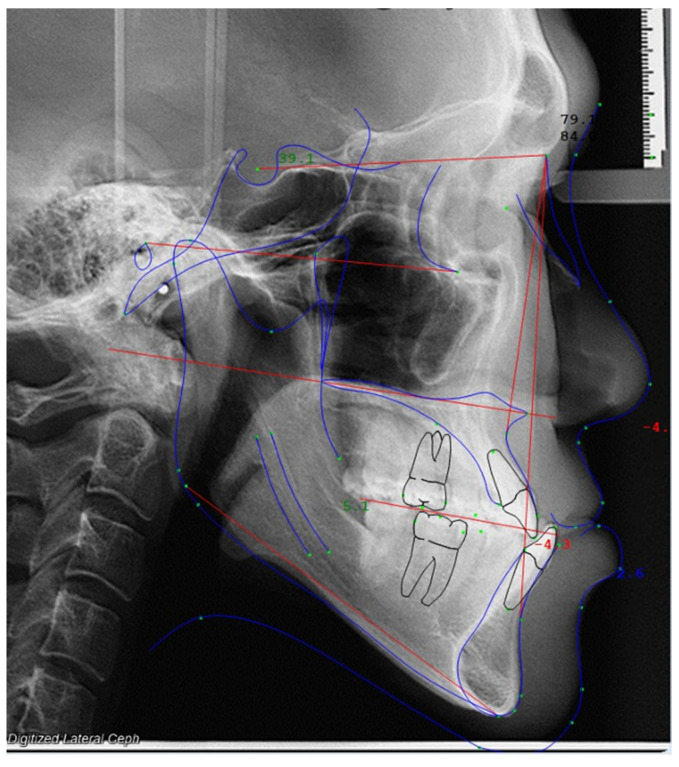
Cephalometric analysis in Dolphin Imaging software.

The lateral cephalograms were traced and digitized by the same investigator using the Dolphin Imaging program version 11.9 (Chatsworth, CA, USA) under standard settings (Figure 1). All lateral cephalograms were digitized by one expert investigator who had board certification in orthodontics and more than five years of clinical experience.

The post-treatment values were input into the treatment simulation module of the Dolphin Imaging software, which subsequently generated a predicted treatment outcome. The values of soft tissue changes for both the actual post-treatment and the predicted treatment outcomes were automatically assessed by the Dolphin measurement function.

**Figure 2:**
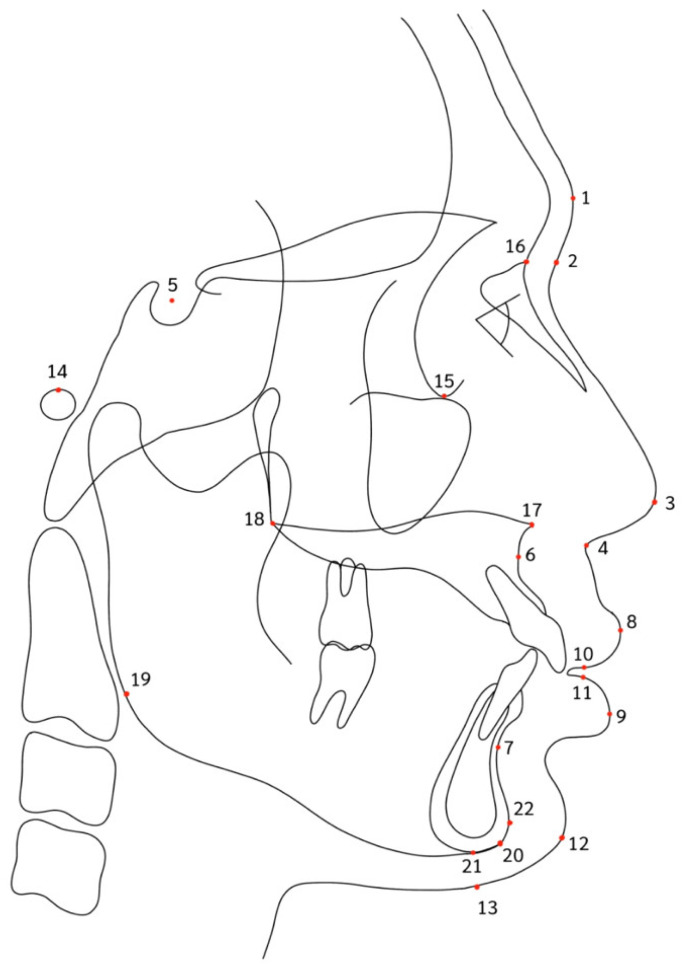
Cephalometric landmarks in this study.

A total of 22 cephalometric landmarks, which included 12 hard tissue and 10 soft tissue landmarks, were selected for both linear and angular measurements (Figure 2 and Table 2). Cephalometric analysis was conducted in accordance with Steiner’s analysis [14], McNamara analysis [15], Holdaway soft tissue analysis [16], and a study by Nuntasukkasame et al. [17] (Table 3). The values of soft tissue changes in the actual post-treatment and predicted treatment outcomes were automatically documented using the measurement function in the Dolphin Imaging software. The differences between the predicted and actual soft tissue changes were calculated by subtracting the predicted values from the actual values. Positive values indicated that the actual values exceeded the predicted values, whereas negative values indicated the opposite.

### 2.3. Statistical Analysis

Statistical analysis of the data was conducted utilizing SPSS statistical software, version 26 (IBM Corp., Armonk, NY, USA). The distribution of data was evaluated using the Shapiro–Wilk test, which confirmed a normal distribution for all variables examined in this study. A paired *t*-test was employed to evaluate the statistical differences between the predicted and actual treatment outcomes for the parameters under investigation. A significance level of 0.05 was established to determine the statistical significance.

### 2.4. Quality Control

All measurements were conducted in a blinded manner, wherein the examiner remained unaware of the treatment group associated with the lateral cephalograms. Intraoperative reliability was evaluated using the intraclass correlation coefficient based on a randomly selected sample of 25 lateral cephalograms obtained after a 2-week interval. Upon comparison of the initial and subsequent measurements using an independent *t*-test, no statistically significant differences were observed between the two sets (*p* < 0.05). Furthermore, the intraclass correlation coefficient, which exceeded 0.92, indicated excellent reliability. Notably, no systematic error was detected for any variable in the paired *t*-test (*p* > 0.05). Random errors were assessed using the Dahlberg formula, which revealed a range of 0.10–0.13 mm for linear cephalometric measurements. These random errors were deemed acceptable.

## 3. Results

### 3.1. Non-Extraction Group

Comparisons between the actual and predicted values in the non-extraction group are shown in Table 4. The facial and upper lip parameters were not significantly different. However, significant differences were found in three parameters of the lower lip: the lower lip sulcus depth (−0.43 mm, *p* = 0.025), the lower lip to the E-plane (−0.44 mm, *p* = 0.001), and the lower lip to the H-line (−0.31 mm, *p* = 0.003), whereas the lower lip length showed no significant differences. The negative values indicated that the soft tissue prediction from the Dolphin Imaging software presented a more anterior position of the lower lip than the actual condition.

### 3.2. Extraction Group

The results of the extraction group are shown in Table 5. Significant differences between the actual and predicted values were found in both the upper and lower lip parameters. The four upper lip parameters included the upper lip sulcus depth (−0.36 mm, *p* = 0.001), the upper lip to the E-plane (−0.39 mm, *p* = 0.001), the upper lip thickness at the A-point (0.97 mm, *p* = 0.002), and the upper lip thickness at the vermillion border (1.01 mm, *p* = 0.004). These indicated that the upper lip prediction from Dolphin Imaging software presented more protrusion and thinner lip thickness at both the A-point and the vermillion border than the actual condition. Three lower lip parameters included the lower lip sulcus depth (−0.35 mm, *p* = 0.017), the lower lip to the E-plane (−0.58 mm, *p* < 0.001), and the lower lip to the H-line (−0.50 mm, *p* = 0.001). These indicated that the lower lip prediction from Dolphin Imaging software presented more protrusion than the actual condition. However, the upper and lower lip lengths, as well as facial parameters, were not significantly different.

### 3.3. Orthodontic Treatment Combined with Orthognathic Surgery

In the orthognathic surgery group, significant differences between the actual and predicted values were found in all parameter categories that included the facial soft tissue and the upper and lower lips (Table 6). The two facial parameters included the facial contour angle (0.95 mm, *p* = 0.002) and chin thickness (0.60 mm, *p* = 0.004). These indicated that the facial soft tissue prediction from Dolphin Imaging software presented more concavity and thinner chin thickness than the actual condition. Two upper lip parameters included the upper lip sulcus depth (−0.55 mm, *p* = 0.039) and the upper lip to the E-plane (−0.51 mm, *p* = 0.002). These indicated that the upper lip prediction from the Dolphin Imaging software presented more protrusion than the actual condition. Three lower lip parameters included the lower lip sulcus depth (0.55 mm, *p* = 0.048), the lower lip to the E-plane (0.68 mm, *p* < 0.001), and the lower lip to the H-line (0.63 mm, *p* < 0.001). These indicated that the lower lip prediction from the Dolphin Imaging software presented more retrusion than the actual condition. However, there were no significant differences in the facial heights and lip lengths.

## 4. Discussion

Orthodontists commonly encounter inquiries concerning potential alterations to the facial profile that result from a specific treatment plan. Consequently, it is essential to ensure accuracy in predicting the treatment results of soft tissue changes. This accuracy assists orthodontists in formulating optimal treatment plans and providing insights into the final appearance of patients, thereby enhancing patient understanding and satisfaction. Nevertheless, discrepancies between the predicted soft tissue response after orthodontic treatment and the actual outcome are a notable concern. Hence, this study assessed the accuracy of digital software in predicting soft tissue changes following orthodontic interventions among patients who underwent treatments that included non-extraction, extraction, and orthodontic treatment combined with orthognathic surgery.

The accuracy of digital software predictions emerges as a crucial determinant in evaluating and predicting a post-treatment soft tissue profile when formulating an effective treatment plan. In this study, the non-extraction group showed that the lower lip position was simulated to be 0.3–0.4 mm more anteriorly than the actual outcome. This finding contradicted a previous study [12], in which only the vertical placement of the lower lip was simulated to appear more inferiorly (about 1.2 mm) than its actual position. The inconsistent results may be attributed to the presence of lower incisor crowding observed in non-extraction cases in our study, which was corrected through the proclination of the lower incisors. A previous study reported that a slight increase in lower lip thickness and protrusion was observed in correlation with an increase in incisal inclination [18]. In addition, the low accuracy in predicting the lower lip could be attributed to several factors, including the flexibility and susceptibility of the lower lip to the impact of incisor position and angulations. Other factors include soft tissue thickness and tonicity, as well as perioral musculature and underlying muscle attachments [19,20].

In the extraction group, orthodontic treatments possess the ability to influence the facial profile and aesthetic aspects of a patient, especially in scenarios involving extractions and substantial anterior retraction [21,22]. The extraction of premolars may potentially lead to increased lip retrusion compared to treatments in non-extraction cases [23]. Differences in the accuracy of lip position prediction were found between the investigations. This study revealed that the upper and lower lip responses after extraction were notably less (0.3–0.5 mm) than the digitally predicted responses when the incisors underwent retraction. Consistent with a previous study [12], the extraction cases predicted a significantly more protruded horizontal position of both lips than what was observed, while the vertical position of the lip remained unchanged. In contrast, the study conducted by Zhang et al. [10] reported more protrusion and inferior positioning of the lips than depicted in real images. This variation can be attributed to distinctions in the populations studied, as Zhang et al. focused on cases of bimaxillary dentoalveolar protrusion. It was observed that cases with bimaxillary protrusion exhibited a slightly greater degree of soft tissue change compared to patients with maxillary protrusion [24]. This suggested that the vertical position of the lips in patients with bimaxillary dentoalveolar protrusion can be influenced by extraction [25]. However, minimal alterations were noted in our study due to the inclusion of borderline extraction cases [26]. Additionally, there was a robust correlation observed between the movement of incisors and both upper and lower lips [27]. The extent of lower lip movement increased proportionally with the degree of maxillary protrusion [24].

Due to the variability and challenges associated with predicting soft tissue changes post-surgically, it is imperative to assess the accuracy of the Dolphin program. This study revealed significant differences between the actual treatment outcomes and the predicted values within the surgery group. The actual values of chin thickness, facial contour angle, and lower lip parameters exceeded the predicted values by 0.5–1.0 mm. However, the actual post-surgical results of the upper lip exhibited more retrusive positions (about 0.5 mm) than the treatment simulation. This aligns with previous studies that indicated that the lips and chin were the most inaccurately predicted landmarks following orthognathic treatment [7,11,28]. Specifically, upper lip landmarks were more likely to be underestimated, while those surrounding the lower lip and chin area tended to be overestimated in the horizontal plane [8]. The poor accuracy of predicting soft tissue changes in the lip and chin regions may be attributed to factors such as soft tissue thickness, tonicity, perioral musculature, and underlying muscle attachments [2,29]. Additionally, the unstable trait of soft tissue thickness in the chin region is associated with the individual’s body mass index [30]. Consequently, these critical considerations were not integrated into the prediction process, which potentially compromised the overall accuracy and comprehensiveness of the prognostic outcomes.

While the utilization of Dolphin Imaging software has enhanced the capacity of clinicians to anticipate soft tissue profiles following orthodontic treatment, there are certain errors in predicting treatment outcomes with some degree of inaccuracy in specific directions. Numerous studies that compared the software-generated predictions with the actual treatment outcomes revealed noteworthy disparities in the measurements [31,32]. This study demonstrated that the mean differences between the predicted and actual postoperative images were confined within a range of less than 2 mm across various subject groups. Despite the presence of statistically significant differences between the simulated and actual positions of specific points, the majority of these differences were of insufficient magnitude to attain clinical significance [8]. Therefore, the Dolphin Imaging software could be considered an alternative tool to predict soft tissue responses in orthodontic treatment with clinically acceptable accuracy.

This study has some limitations. First, it was conducted using two-dimensional analysis, which limited the measurement of changes in projections or variations in lip length along a fixed plane. To enhance the capability of soft tissue analysis, 3D construction from cone beam computed tomography and a 3D facial scanner is recommended for further study. The incorporation of this advancement into future studies has the potential to significantly enhance the understanding and accuracy of the analyses, which would offer a more comprehensive perspective on orthodontic and surgical planning. Second, the Dolphin software generates a prediction based on a fixed ratio of movement between the soft and hard tissues to simulate changes following treatment. Unfortunately, the changes that occur after treatment are not limited to hard tissue movement alone. Numerous additional factors exert an impact on soft tissue modifications that include thickness, tension, dentofacial morphology, and the measurement technology employed [13,24,33]. Consequently, it is essential to take these factors into account during the prediction process to prevent unrealistic expectations and patient dissatisfaction. Finally, this was a retrospective study. The subjects were not perfectly homogeneous due to different types and magnitudes of dental and skeletal discrepancies. Additional studies are needed to investigate the subtypes of orthognathic surgery for different skeletal discrepancies or facial profiles.

## 5. Conclusions

Soft tissue prediction presented errors that ranged from 0.3 to 1.0 mm for all interventions. The prediction errors in the non-extraction group presented more protrusion of the lower lip than the final actual outcome, while the extraction group presented more protrusion of both the upper and lower lips than the actual outcome. In the orthognathic surgery group, prediction errors were observed in chin thickness, facial contour angle, and in both the upper and lower lip parameters.

## Figures and Tables

**Table 1:** Inclusion and exclusion criteria.

Inclusion Criteria	Exclusion Criteria
(1) Adult subjects within the pre-treatment age of 18–30 years (2) Presence of comprehensive records that included treatment details and lateral cephalometric radiographs for both pre- and post-treatment (3) Good image quality of the lateral cephalograms (4) Availability of lateral cephalograms enabling the identification of specific hard and soft tissue cephalometric landmarks (5) No other pathologies that could affect soft and hard tissue anatomy	(1) History of craniofacial trauma, syndromes, or deformities (2) History of cosmetic or reconstructive facial surgery (3) History of using medications that could impact soft tissue (4) History of tooth extraction unrelated to orthodontic treatment

**Table 2:** Cephalometric landmarks.

Landmark	Definition
1. Soft tissue glabella (G’)	Most prominent point in the sagittal plane between the supraorbital ridges.
2. Soft tissue nasion (N’)	Deepest part of the soft tissue outlines in front of the nasion.
3. Pronasale (P)	Tip of the nose.
4. Subnasale (Sn’)	Junction of the nasal septum and upper lip in the mid-sagittal plane.
5. Sella (S)	Geometric center of the pituitary fossa (sella turcica).
6. A-point (A)	Deepest point on the maxilla below the ANS.
7. B-point (B)	Most posterior point on the bony curve of the mandible above the pogonion.
8. Labialis superior (Ls)	Most anterior point on the outline of the upper lip (vermillion border).
9. Labialis inferior (Li)	Most anterior point on the outline of the lower lip (vermillion border).
10. Stomium superior (Stms)	Lowest midline point on the outline of the upper lip (vermillion border).
11. Stomium inferior (Stmi)	Highest midline point on the outline of the lower lip (vermillion border).
12. Soft tissue pogonion (Pog’)	Most anterior point on the outline of the soft tissue chin.
13. Soft tissue menton (Me’)	Lowest point on the outline of the soft tissue chin.
14. Porion (Po)	Top of the external auditory meatus.
15. Orbitale (Or)	Inferior border of orbit.
16. Nasion (N)	Midpoint of the frontonasal sutures in the midsagittal plane.
17. Anterior nasal spine (ANS)	Anterior point of the maxilla at the base of the nose.
18. Posterior nasal spine (PNS)	Posterior point of the bony hard palate.
19. Gonion (Go)	Most posterior and inferior point on the outline of the angle of the mandible.
20. Gnathion (Gn)	Most anterior and inferior point on the bony chin.
21. Menton (Me)	Lowest point on the symphysis of the mandible.
22. Pogonion (Pog)	Most anterior point on the contour of the bony chin in the midsagittal plane.

**Table 3:** Soft tissue parameters.

Parameter	Operational Definition
Soft tissue chin thickness (mm)	Distance between the hard and soft tissue facial planes at the level of the suprapogonion.
Upper lip to the E-plane (mm)	Distance between the upper lip and the E-plane.
Lower lip to the E-plane (mm)	Distance between the lower lip and the E-plane.
H-Angle (°)	Angle formed between the soft tissue facial plane line and the H-line.
Lower lip to the H-line (mm)	Distance between the lower lip and the H-line.
Soft tissue subnasale to the H-line (mm)	Measurement from the subnasale to the H-line.
Upper lip thickness at the vermillion border (mm)	Dimension between the vermillion point and the labial surface of the maxillary incisor.
Upper lip thickness at the A-point (mm)	Dimension measured approximately 3 mm below point A and the drape of the upper lip.
Upper lip sulcus depth (mm)	Length between the sulcus of the upper lip and a perpendicular line traced from the vermillion plane to the Frankfurt plane.
Lower lip sulcus depth (mm)	Measurement determined between the vermillion border of the lower lip and the H-line at the point of greatest convexity.
NLA (nasolabial angle)	Angle generated by a line drawn through the middle of the nostril aperture, intersecting the subnasale, and a line drawn perpendicular to the Frankfurt horizontal.
Facial contour angle (°)	Angle generated by intersecting the G’-Sn and Sn-Pog’ planes.
UFH (mm)	Distance from the midpoint of the eye to the subnasale.
LFH (mm)	Distance from the subnasale to the soft tissue menton.
ULL (mm)	Distance from the Sn-Stms.
LLL (mm)	Distance from the Stmi-Me’.

UFH, upper facial height; LFH, lower facial height; ULL, upper lip length; LLL, lower lip length.

**Table 4:** Comparisons of the soft tissue parameters between actual values and predicted values in non-extraction.

Soft Tissue Parameters	Actual Values (Mean ± SD)	Predicted Values (Mean ± SD)	Differences (Mean ± SD)	*p* Value
**Facial parameters**				
Facial contour angle (°)	−7.87 ± 0.88	−7.42 ± 0.85	−0.45 ± 0.31	0.153
UFH (mm)	44.39 ± 0.71	44.67 ± 0.67	−0.28 ± 0.50	0.582
LFH (mm)	66.99 ± 0.82	66.30 ± 0.83	0.69 ± 0.40	0.098
Chin thickness	11.79 ± 0.29	11.90 ± 0.35	−0.12 ± 0.16	0.486
**Upper lip parameters**				
Nasolabial angle (°)	96.47 ± 1.88	98.29 ± 1.96	−1.82 ± 1.14	0.125
Subnasale to the H-line (mm)	9.03 ± 0.36	8.66 ± 0.32	0.37 ± 0.29	0.225
Upper lip sulcus depth (mm)	5.32 ± 0.21	5.13 ± 0.23	0.19 ± 0.17	0.267
H-angle (°)	18.11 ± 0.66	18.23 ± 0.62	−0.12 ± 0.49	0.809
Upper lip to the E-plane (mm)	0.21 ± 0.04	0.26 ± 0.05	−0.05 ± 0.04	0.191
U-Lip thickness at the A-point (mm)	13.60 ± 0.47	14.26 ± 0.48	−0.66 ± 0.39	0.110
U-Lip thickness at the vermillion border (mm)	12.90 ± 0.42	13.00 ± 0.33	−0.09 ± 0.35	0.785
Upper lip length (mm)	22.10 ± 0.37	21.82 ± 0.43	0.28 ± 0.23	0.237
**Lower lip parameters**				
Lower lip sulcus depth (mm)	3.82 ± 0.14	4.25 ± 0.12	−0.43 ± 0.18	0.025 *
Lower lip length (mm)	45.05 ± 0.70	44.21 ± 0.68	0.84 ± 0.58	0.165
Lower lip to the E-plane (mm)	1.52 ± 0.16	1.96 ± 0.20	−0.44 ± 0.12	0.001 **
Lower lip to the H-line (mm)	1.31 ± 0.14	1.62 ± 0.17	−0.31 ± 0.09	0.003 **

UFH, upper facial height; LFH, lower facial height. * *p* < 0.05, ** *p* < 0.01.

**Table 5:** Comparisons of the soft tissue parameters between actual values and predicted values in extraction.

Soft Tissue Parameters	Actual Values (Mean ± SD)	Predicted Values (Mean ± SD)	Differences (Mean ± SD)	*p* Value
**Facial parameters**				
Facial contour angle (°)	9.66 ± 0.34	9.62 ± 0.31	0.04 ± 0.32	0.386
UFH (mm)	44.42 ± 0.60	44.82 ± 0.66	−0.40 ± 0.27	0.272
LFH (mm)	64.90 ± 1.24	64.76 ± 1.14	0.14 ± 0.06	0.627
Chin thickness	11.50 ± 0.24	11.49 ± 0.31	0.01 ± 0.24	0.871
**Upper lip parameters**				
Nasolabial angle (°)	103.09 ± 1.99	101.25 ± 1.86	1.83 ± 1.18	0.134
Subnasale to the H-line (mm)	8.14 ± 0.34	8.20 ± 0.34	−0.06 ± 16	0.716
Upper lip sulcus depth (mm)	3.92 ± 0.15	4.28 ± 0.15	−0.36 ± 0.10	0.001 **
H-angle (°)	18.66 ± 0.34	18.54 ± 0.30	0.12 ± 0.20	0.564
Upper lip to the E-plane (mm)	0.20 ± 0.13	0.59 ± 0.17	−0.39 ± 0.03	0.001 **
U-Lip thickness at the A-point (mm)	16.07 ± 0.32	15.10 ± 0.33	0.97 ± 0.28	0.002 **
U-Lip thickness at the vermillion border (mm)	14.48 ± 0.48	13.47 ± 0.50	1.01 ± 0.35	0.004 **
Upper lip length (mm)	21.42 ± 0.48	21.23 ± 0.41	0.19 ± 0.33	0.257
**Lower lip parameters**				
Lower lip sulcus depth (mm)	3.47 ± 0.16	3.12 ± 0.17	−0.35 ± 0.09	0.017 *
Lower lip length (mm)	43.96 ± 0.77	43.85 ± 0.67	0.11 ± 0.50	0.813
Lower lip to the E-plane (mm)	1.95 ± 0.29	2.53 ± 0.37	−0.58 ± 0.13	<0.001 ***
Lower lip to the H-line (mm)	1.68 ± 0.21	2.18 ± 0.28	−0.50 ± 0.12	0.001 **

UFH, upper facial height; LFH, lower facial height. * *p* < 0.05, ** *p* < 0.01, *** *p* < 0.001.

**Table 6:** Comparisons of the soft tissue parameters between actual values and predicted values in orthodontic treatment combined with orthognathic surgery.

Soft Tissue Parameters	Actual Values (Mean ± SD)	Predicted Values (Mean ± SD)	Differences (Mean ± SD)	*p* Value
**Facial parameters**				
Facial contour angle (°)	8.55 ± 0.42	7.60 ± 0.38	0.95 ± 0.25	0.002 **
UFH (mm)	25.07 ± 0.74	25.50 ± 0.79	0.43 ± 0.11	0.381
LFH (mm)	68.44 + 0.86	68.85 ± 0.95	−0.41 ± 0.42	0.271
Chin thickness	12.38 ± 0.24	11.78 ± 0.23	0.60 ± 0.19	0.004 **
**Upper lip parameters**				
Nasolabial angle (°)	100.46 ± 1.49	100.10 ± 1.50	0.36 ± 1.06	0.735
Subnasale to the H-line (mm)	7.51 ± 0.17	7.30 ± 0.15	0.21 ± 0.14	0.054
Upper lip sulcus depth (mm)	3.68 ± 0.33	4.23 ± 0.35	−0.55 ± 0.21	0.039 *
H-angle (°)	14.65 ± 0.50	14.34 ± 0.55	0.32 ± 0.27	0.250
Upper lip to the E-plane (mm)	0.25 ± 0.08	0.66 ± 0.11	−0.51 ± 0.14	0.002 **
U-Lip thickness at the A-point (mm)	13.65 ± 0.29	13.78 ± 0.29	−0.14 ± 0.09	0.486
U-Lip thickness at the vermillion border (mm)	12.79 ± 0.36	12.86 ± 0.33	−0.17 ± 0.04	0.102
Upper lip length (mm)	22.45 ± 0.47	22.42 ± 0.41	0.03 ± 0.15	0.645
**Lower lip parameters**				
Lower lip sulcus depth (mm)	4.26 ± 0.26	3.71 ± 0.34	0.55 ± 0.28	0.048 *
Lower lip length (mm)	45.06 ± 0.60	44.65 ± 0.63	0.41 ± 0.12	0.276
Lower lip to the E-plane (mm)	2.61 ± 0.41	1.93 ± 0.38	0.68 ± 0.12	<0.001 ***
Lower lip to the H-line (mm)	2.72 ± 0.39	2.09 ± 0.35	0.63 ± 0.14	<0.001 ***

UFH, upper facial height; LFH, lower facial height. * *p* < 0.05, ** *p* < 0.01, *** *p* < 0.001.

## Data Availability

Data are available upon request.
